# Supplementary material for: Wastewater surveillance of SARS-CoV-2 from aircraft to citywide monitoring
Source: Nat Commun. 2025 Jun 2;16:5125. doi: 10.1038/s41467-025-60490-1 (PMC12130461; doi:10.1038/s41467-025-60490-1)
Supplement: Supplementary file 1 — Supplementary Information [file 41467_2025_60490_MOESM1_ESM.pdf]

SUPPLEMENTARY MATERIAL

Wastewater surveillance of SARS-CoV-2 from aircraft to citywide monitoring

Mariel Perez-Zabaleta[a,1], Carlo Berg[b], Neus Latorre-Margalef[b], Isaac Owusu-Agyeman[a], Ayda Kiyar[a], Helene Botnen[c], Caroline Schönning[c], Luisa W. Hugerth[d*], Zeynep Cetecioglu[a,*]

[a] Department of Industrial Biotechnology, School of Engineering Sciences in Chemistry Biotechnology and Health, AlbaNova University Center, KTH Royal Institute of Technology, Stockholm, Sweden.

[b] Public Health Agency of Sweden, Department of Microbiology, Solna, Sweden

[c] Public Health Agency of Sweden, Department of Communicable Disease Control and Health Protection, Solna, Sweden

[d] Department of Medical Biochemistry and Microbiology, Science for Life Laboratory, Uppsala University, Uppsala, Sweden.

*Co-corresponding authors: luisa.hugerth@scilifelab.se (L. W. Hugerth) and zeynepcg@kth.se (Z. Cetecioglu)

[1] Present address: National Food Institute, Technical University of Denmark, Kongens Lyngby, Denmark.

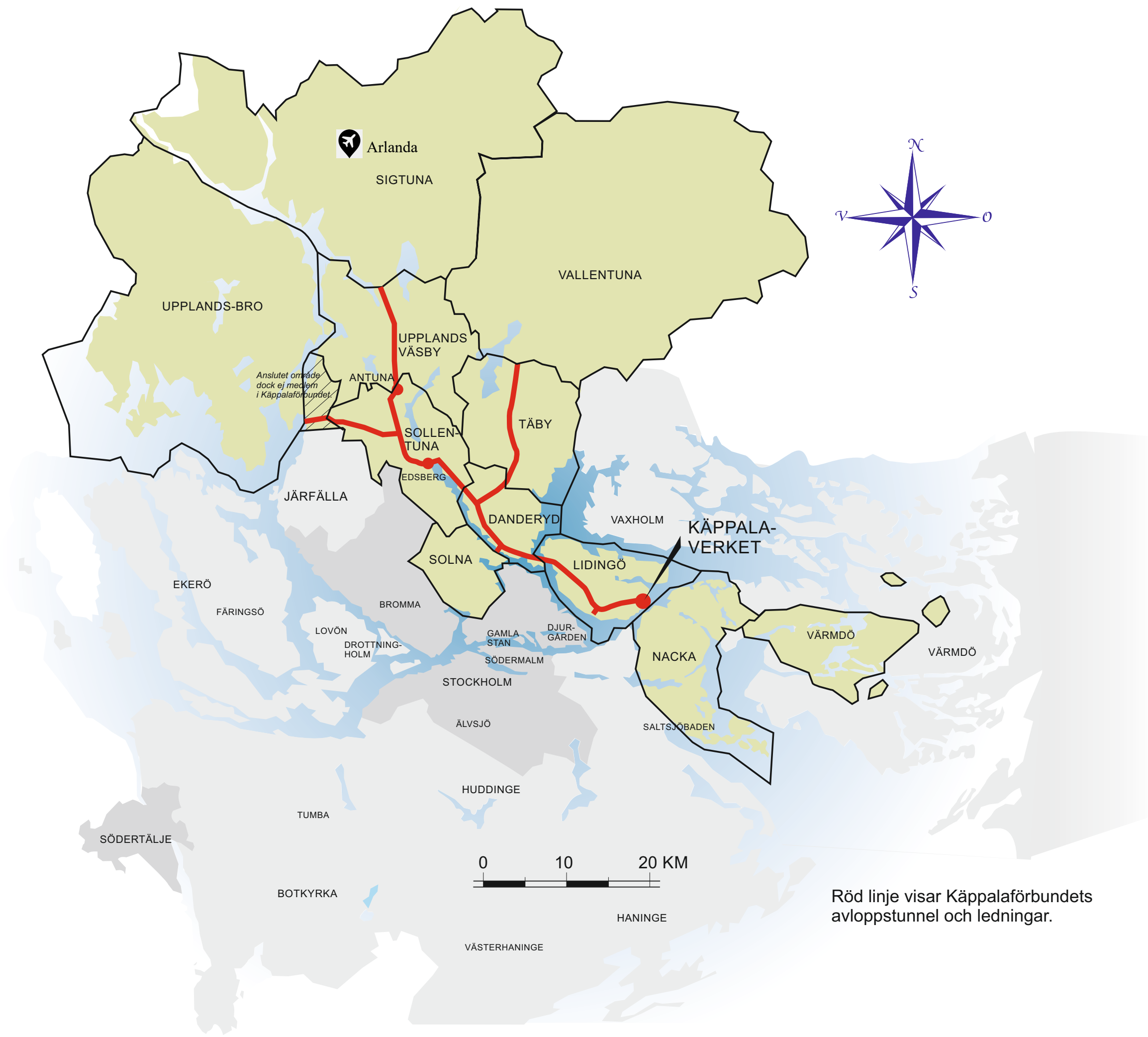

Figure S1. Regions covered by Käppala WWTP. Latitude and longitude: 59.35, 18.23. Information provided by the wastewater treatment plant and adapted from Perez-Zabaleta M et. al, Long-term SARS-CoV-2 surveillance in the wastewater of Stockholm: What lessons can be learned from the Swedish perspective? Sci Total Environ. 2023, PMC9640212, with permission. All airport wastewaters including Arlanda Airport Terminal 5 and Måby station (covering the airport region) go to Käppala WWTP. Käppala WWTP has only one inlet.

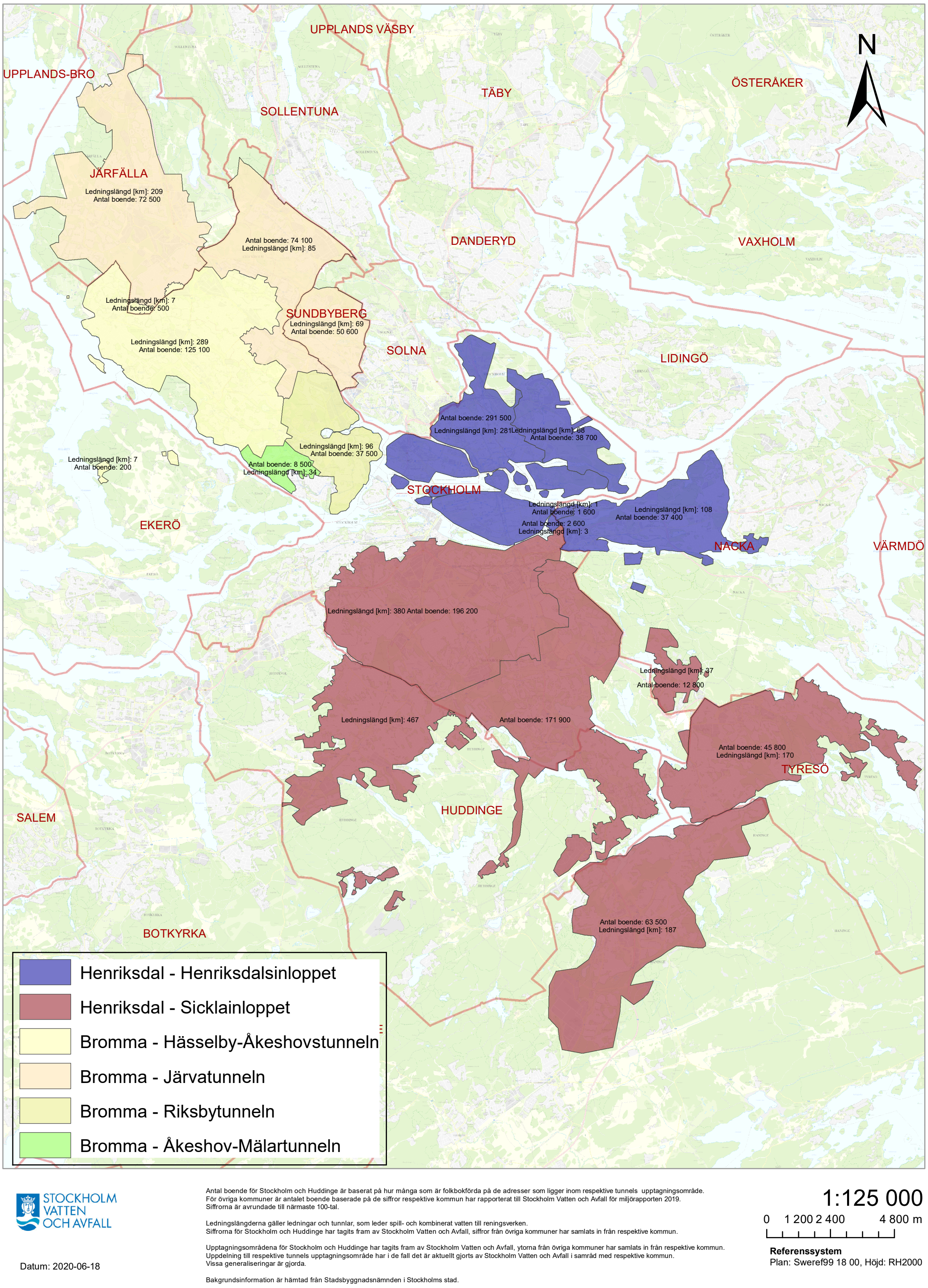

Figure S2. Regions covered by Henriksdal WWTP and Bromma WWTP. Latitude and longitude for Henriksdal WWTP: 59.31, 18.11 and Bromma WWTP: 59.34, 17.93. Information provided by Stockholm Vatten och Avfall and reproduced with permission from Perez-Zabaleta M et. al, Long-term SARS-CoV-2 surveillance in the wastewater of Stockholm: What lessons can be learned from the Swedish perspective? Sci Total Environ. 2023, PMC9640212. Henriksdal WWTP has two inlets: Henriksdal (purple) and Sickla (crimson red). Bromma has four inlets but three were sampled: Hässelby (light yellow), Järva (beige) and Riksby (yellow).

METHODS: Comparison of three primers targeting the nucleocapsid gene and TaqMan vs SYBRGreen methods.

For the evaluation of primer sets, two types of samples were selected: one with high Ct values (low viral levels, Aircraft) and one with low Ct values (high viral levels, Käppala). The three sets of primers and probes (N1, N2 and N3) were ordered from Sigma-Aldrich (Merck KGaA, Germany) and the primers sequences were according to Lu et al., (2020). For the evaluation of the methods, SYBRGreen and TaqMan, aircraft samples were selected since it was desired to study the sensitivity of the methods, which is crucial for detecting low levels of SARS-CoV-2 RNA.

Quantification was performed using 5 µL Reliance One-Step Multiplex RT-qPCR Supermix Kit (Bio-Rad), 5 µL of RNA template, 2 µL of primers (final concentration of 500 nM), 0.25 µL probe (final concentration of 125 nM), 1 µL BSA and the rest was nuclease-free water for a total reaction volume of 20 µL. Thermal cycling (50 °C 10 min, 95 °C 10 min, followed by 45 cycles of 95 °C 10 s, 60 °C for 30 s) on an Applied Biosystems Quantstudio 3 (Fisher Scientific™, Sweden) machine was performed. Two tap water samples were used as negative controls in each extraction set (extraction of 16 samples) and subsequently analysed by qPCR. Nuclease-free water was also used as negative control for qPCR analysis. SARS-CoV-2 DNA (2019-nCoV_N_Positive Control, IDT, Cat. 10006625), and a constructed plasmid containing the appropriate target for PMMoV (IDT, custom MiniGene 25-500 bp) were used as positive controls and to create the standard curves. Cross-plate controls with known SARS-CoV-2 and PMMoV concentrations were used in each qPCR analysis for reference and quality control.

To compare N1, N2 and N3 primers, the third calculation method has been applied (N-gene copies per 100 mL wastewater) because unnormalized data was preferred to have a clearer comparison of raw data. In this calculation approach, Ct values of either N1, N2 or N3 were

converted to gene copy number per reaction (copies per 20 µL of reaction volume) using the corresponding standard curves. Then, the respective dilutions (5µL template, 80 µL RNA extracted and 40 mL initial sample) were considered to calculate the gene copy number per mL of wastewater. The data was expressed as gene copy numbers per 100 mL of wastewater.

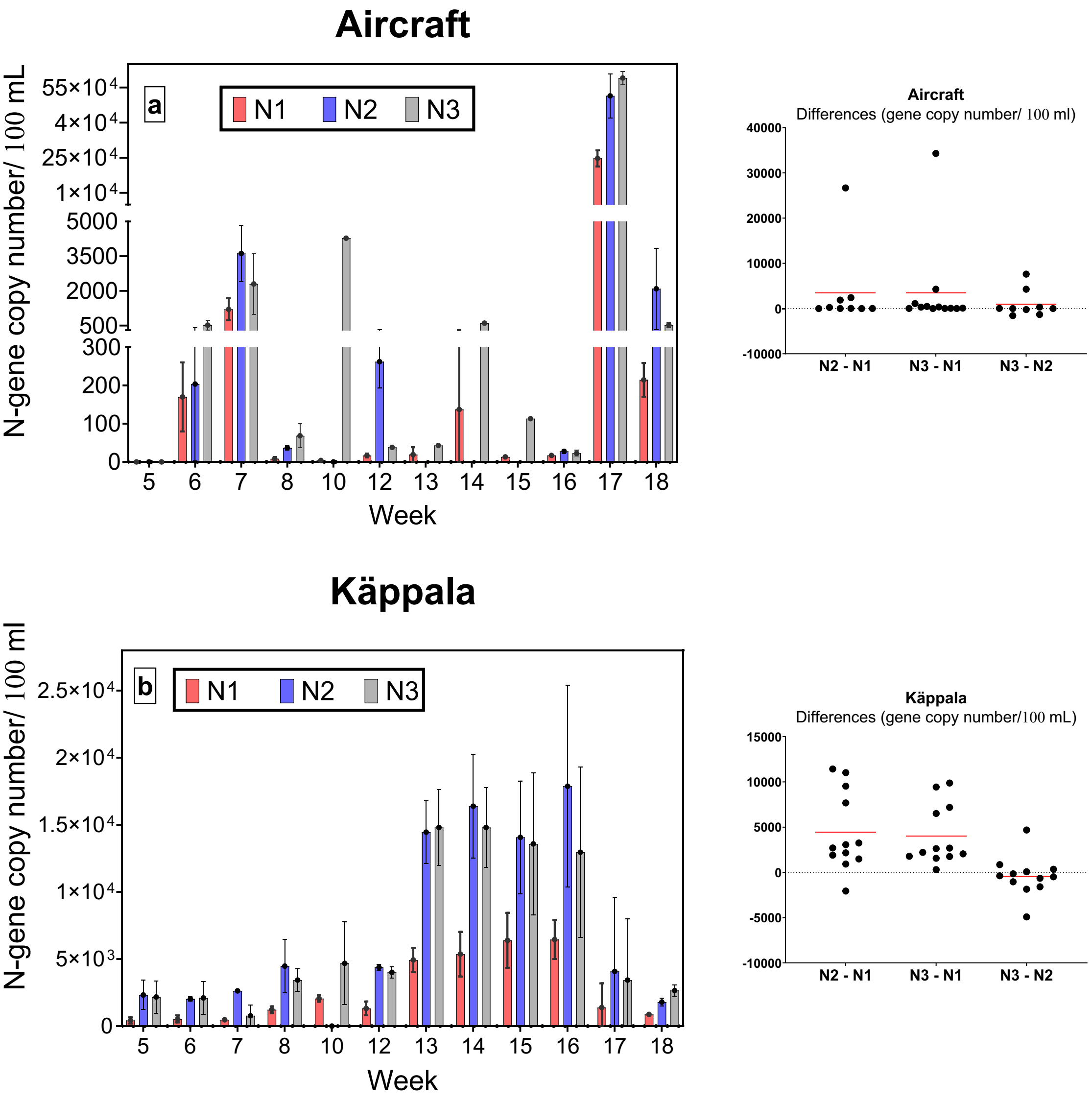

Figure S3. Comparison of N1, N2 and N3 primers for SARS-CoV-2 detection using samples from (a) aircraft and (b) Käppala. One-way analysis of variance (ANOVA) was used to determine whether there were any statistically significant differences among the three tested primers. Käppala was used as a reference as these samples had low Ct values throughout the monitoring period. no significant difference between the N2 and N3 primer sets when the Käppala samples were analysed, and the N1 primer set was different from both the N2 and N3 primer sets. Data are presented as mean values +/- standard deviation (SD), n= 12 samples (weeks).

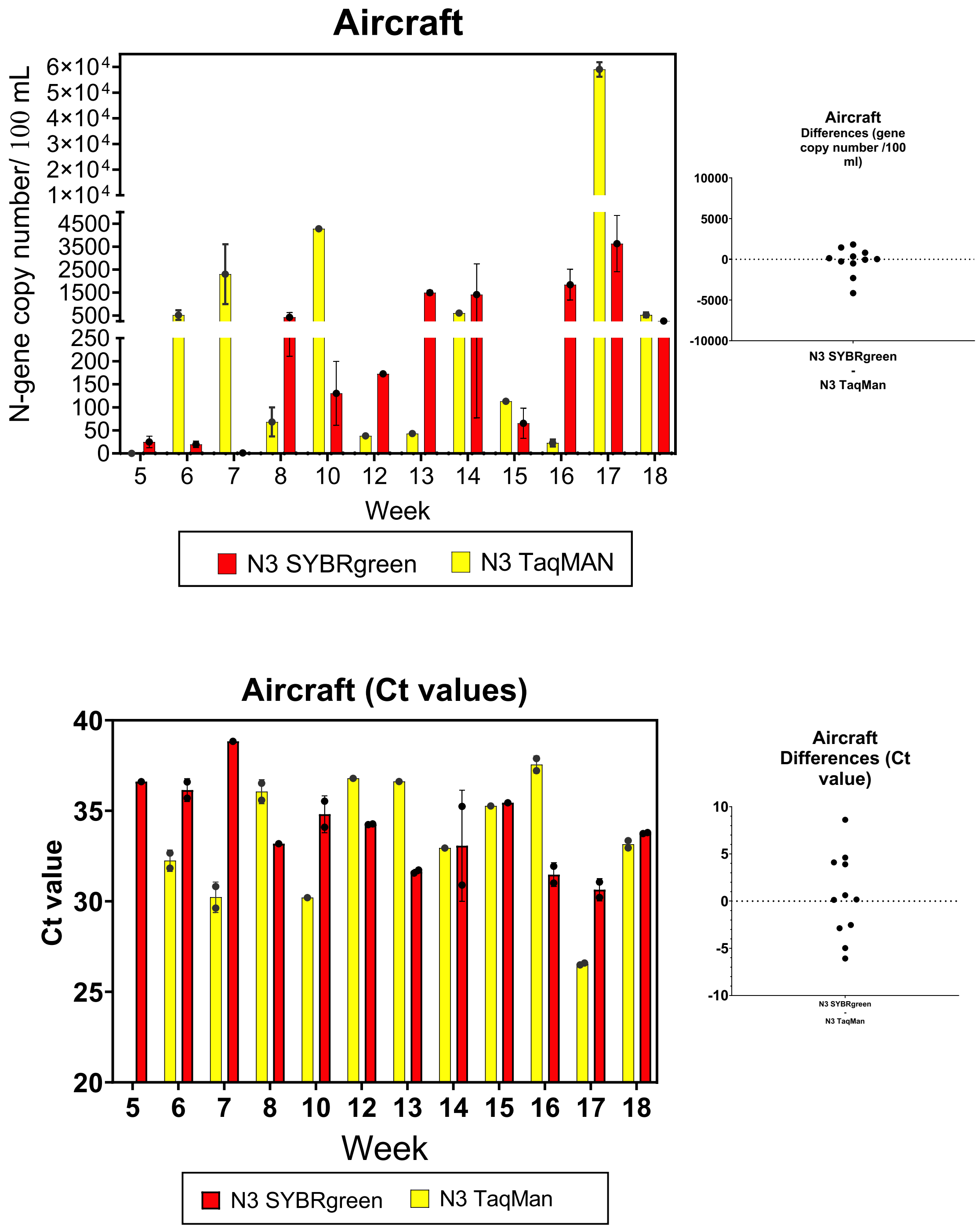

Figure S4. Comparison of SYBRGreen and TaqMan methods using N3 primers for SARS-CoV-2 detection in aircraft samples. One-way analysis of variance (ANOVA) was used to determine whether there were any statistically significant differences among the tested method. No significant difference between the SYBRGreen and TaqMan method were found. Data are presented as mean values +/- standard deviation (SD), n= 12 samples (weeks).

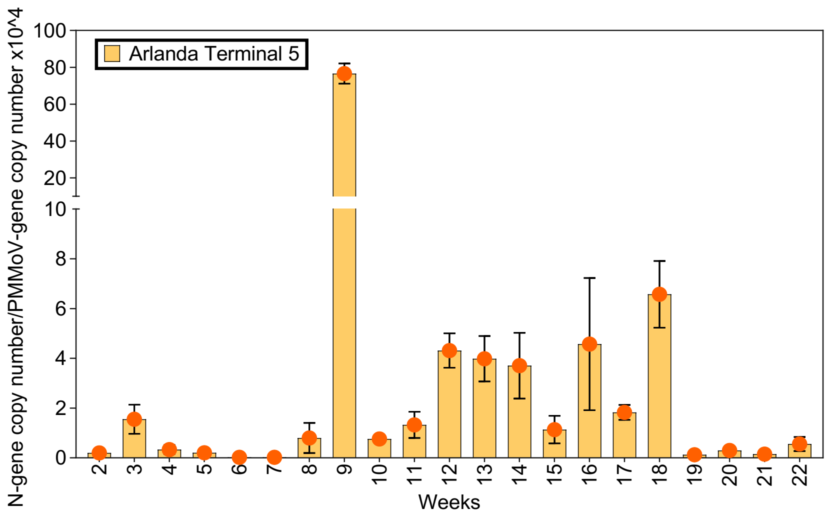

Figure S5. SARS-CoV-2 content presented as N-gene copy number per PMMoV gene copy of Stockholm Arlanda Airport Terminal 5. Two biological replicates and two technical replicates were analysed for each data point. Data are presented as mean values +/- standard deviation (SD), n= 21 samples (weeks).

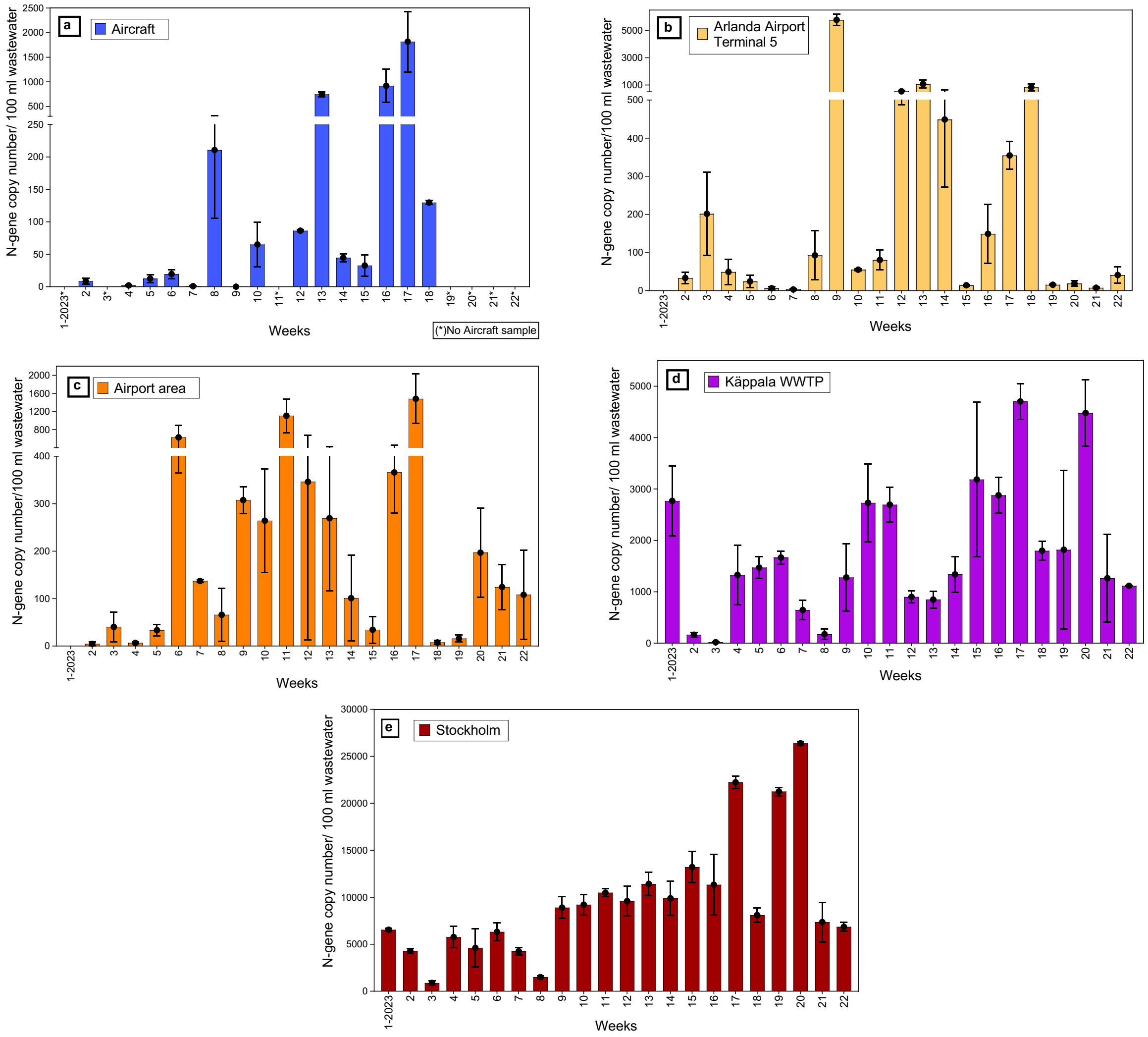

Figure S6. SARS-CoV-2 content per 100 mL raw wastewater. (a) Aircraft (blue) n= 15 samples (weeks). (b) Arlanda Airport Terminal 5 (yellow), n= 21 samples (weeks). (c) Airport area (orange), n= 21 samples (weeks) (d) Käppala WWTP (purple), n= 22 samples (weeks) (e) Stockholm (brown), n= 22 samples (weeks). (*) No aircraft sample in week 3 and 11. Two biological replicates and two technical replicates were analysed for each sample. Data are presented as mean values +/- standard deviation (SD).

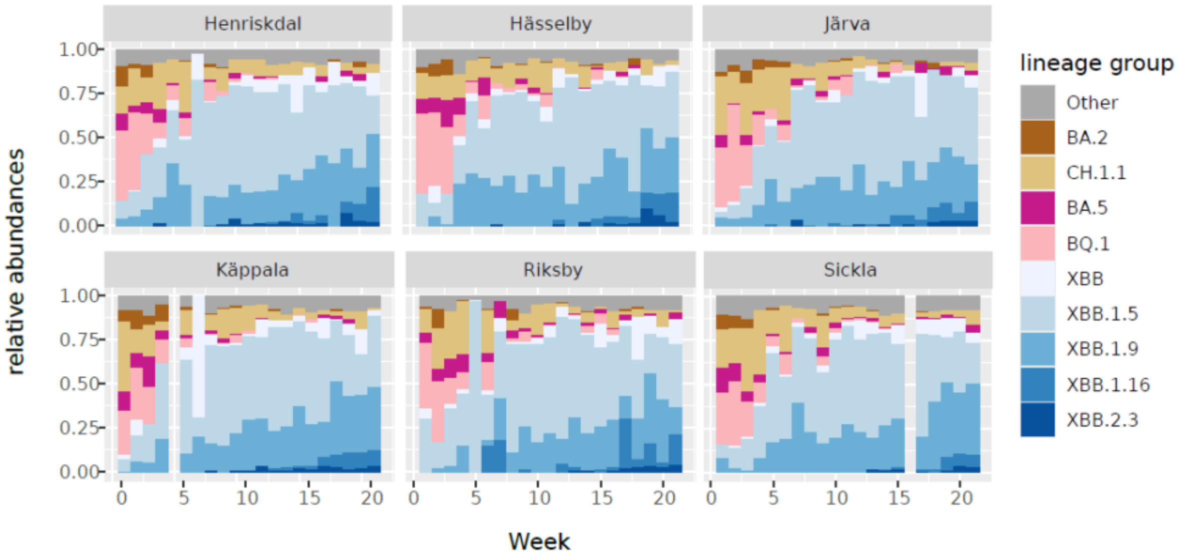

Figure S7: Detected lineage groups for each week in each of the six WWTP inlets in the Stockholm metropolitan area.

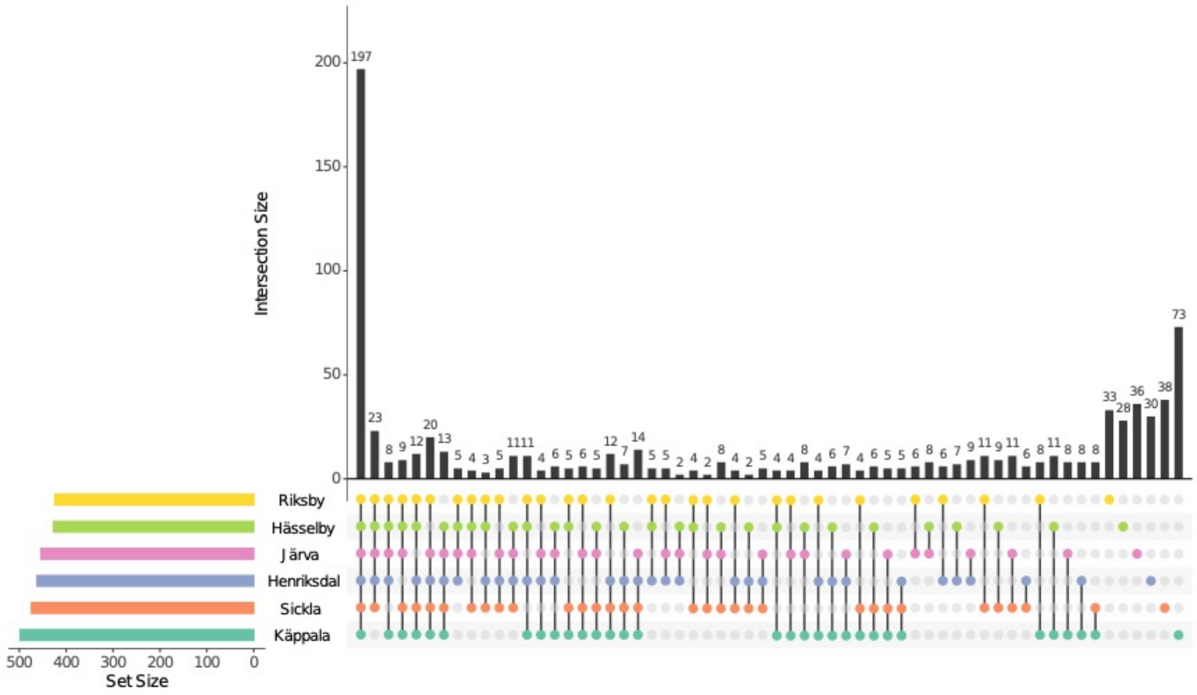

Figure S8: Upset plot of each of the six wastewater inlets in the Stockholm metropolitan area. An Upset plot shows a horizontal bar plot of the total number of lineages found at each site and on the vertical bar plot the number of lineages found at each combination of sites. Combinations are depicted as coloured dots corresponding to the sites on the left.

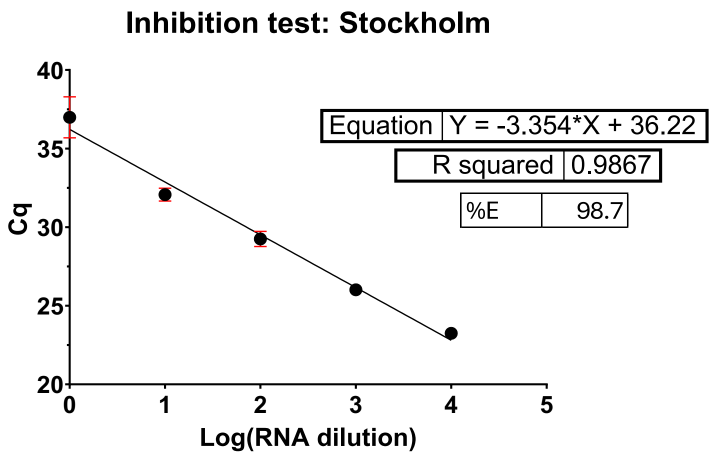

Figure S9: Inhibition testing on a pooled sample from Stockholm. The samples were subjected to a 10-fold serial dilution. Calibration parameters, including R^2 correlation, slope, and qPCR efficiency, were calculated to assess the accuracy and reliability of the inhibition testing
