# Supplementary material for: Wastewater surveillance of SARS-CoV-2 from aircraft to citywide monitoring
Source: Nat Commun. 2025 Jun 2;16:5125. doi: 10.1038/s41467-025-60490-1 (PMC12130461; doi:10.1038/s41467-025-60490-1)
Supplement: Supplementary file 3 — Reporting Summary [file 41467_2025_60490_MOESM3_ESM.pdf]

nature portfolio

Corresponding author(s): Luisa W. Hugerth and Zeynep Cetecioglu

Last updated by author(s): 2025(05/08

Reporting Summary

Nature Portfolio wishes to improve the reproducibility of the work that we publish. This form provides structure for consistency and transparency in reporting. For further information on Nature Portfolio policies, see our Editorial Policies and the Editorial Policy Checklist.

Statistics

For all statistical analyses, confirm that the following items are present in the figure legend, table legend, main text, or Methods section.

n/a	Confirmed	
☐	☒	The exact sample size (*n*) for each experimental group/condition, given as a discrete number and unit of measurement
☐	☒	A statement on whether measurements were taken from distinct samples or whether the same sample was measured repeatedly
☐	☒	The statistical test(s) used AND whether they are one- or two-sided<br>*Only common tests should be described solely by name; describe more complex techniques in the Methods section.*
☐	☒	A description of all covariates tested
☐	☒	A description of any assumptions or corrections, such as tests of normality and adjustment for multiple comparisons
☐	☒	A full description of the statistical parameters including central tendency (e.g. means) or other basic estimates (e.g. regression coefficient) AND variation (e.g. standard deviation) or associated estimates of uncertainty (e.g. confidence intervals)
☐	☒	For null hypothesis testing, the test statistic (e.g. *F*, *t*, *r*) with confidence intervals, effect sizes, degrees of freedom and *P* value noted<br>*Give P values as exact values whenever suitable.*
☒	☐	For Bayesian analysis, information on the choice of priors and Markov chain Monte Carlo settings
☐	☒	For hierarchical and complex designs, identification of the appropriate level for tests and full reporting of outcomes
☐	☒	Estimates of effect sizes (e.g. Cohen's *d*, Pearson's *r*), indicating how they were calculated

Our web collection on statistics for biologists contains articles on many of the points above.

Software and code

Policy information about availability of computer code

Data collection	not applicable
Data analysis	Raw sequencing reads went through Torrent Suit AmpliSeq SARS-CoV-2 . The resulting BAM files were post-processed with Freyja v.1.4.8

For manuscripts utilizing custom algorithms or software that are central to the research but not yet described in published literature, software must be made available to editors and reviewers. We strongly encourage code deposition in a community repository (e.g. GitHub). See the Nature Portfolio guidelines for submitting code & software for further information.

Data

Policy information about availability of data

All manuscripts must include a data availability statement. This statement should provide the following information, where applicable:

- Accession codes, unique identifiers, or web links for publicly available datasets
- A description of any restrictions on data availability
- For clinical datasets or third party data, please ensure that the statement adheres to our policy

Data Sharing
Data from wastewater surveillance is accessible at the European Nucleotide Archive ENA under Project: PRJEB61810 and ERP146892. As for the occurrence data of COVID-19 clinical cases confirmed by PCR, it is publicly available through http://fohm-app.folkhalsomyndigheten.se/Folkhalsodata/pxweb/en/A_Folkhalsodata/A_Folkhalsodata__H_Sminet__covid19__testdata/PCRtest.px/. Sequences from clinical cases are part of the national genomic surveillance and are available at

GISAID searching by Sweden.

Research involving human participants, their data, or biological material

Policy information about studies with human participants or human data. See also policy information about sex, gender (identity/presentation), and sexual orientation and race, ethnicity and racism.

Reporting on sex and gender	not applicable for this research
Reporting on race, ethnicity, or other socially relevant groupings	not applicable for this research
Population characteristics	not applicable for this research
Recruitment	not applicable for this research
Ethics oversight	not applicable for this research

Note that full information on the approval of the study protocol must also be provided in the manuscript.

Field-specific reporting

Please select the one below that is the best fit for your research. If you are not sure, read the appropriate sections before making your selection.

☐ Life sciences ☐ Behavioural & social sciences ☒ Ecological, evolutionary & environmental sciences

For a reference copy of the document with all sections, see nature.com/documents/nr-reporting-summary-flat.pdf

Life sciences study design

All studies must disclose on these points even when the disclosure is negative.

Sample size	*Describe how sample size was determined, detailing any statistical methods used to predetermine sample size OR if no sample-size calculation was performed, describe how sample sizes were chosen and provide a rationale for why these sample sizes are sufficient.*
Data exclusions	*Describe any data exclusions. If no data were excluded from the analyses, state so OR if data were excluded, describe the exclusions and the rationale behind them, indicating whether exclusion criteria were pre-established.*
Replication	*Describe the measures taken to verify the reproducibility of the experimental findings. If all attempts at replication were successful, confirm this OR if there are any findings that were not replicated or cannot be reproduced, note this and describe why.*
Randomization	*Describe how samples/organisms/participants were allocated into experimental groups. If allocation was not random, describe how covariates were controlled OR if this is not relevant to your study, explain why.*
Blinding	*Describe whether the investigators were blinded to group allocation during data collection and/or analysis. If blinding was not possible, describe why OR explain why blinding was not relevant to your study.*

Behavioural & social sciences study design

All studies must disclose on these points even when the disclosure is negative.

Study description	*Briefly describe the study type including whether data are quantitative, qualitative, or mixed-methods (e.g. qualitative cross-sectional, quantitative experimental, mixed-methods case study).*
Research sample	*State the research sample (e.g. Harvard university undergraduates, villagers in rural India) and provide relevant demographic information (e.g. age, sex) and indicate whether the sample is representative. Provide a rationale for the study sample chosen. For studies involving existing datasets, please describe the dataset and source.*
Sampling strategy	*Describe the sampling procedure (e.g. random, snowball, stratified, convenience). Describe the statistical methods that were used to predetermine sample size OR if no sample-size calculation was performed, describe how sample sizes were chosen and provide a rationale for why these sample sizes are sufficient. For qualitative data, please indicate whether data saturation was considered, and what criteria were used to decide that no further sampling was needed.*
Data collection	*Provide details about the data collection procedure, including the instruments or devices used to record the data (e.g. pen and paper, computer, eye tracker, video or audio equipment) whether anyone was present besides the participant(s) and the researcher, and whether the researcher was blind to experimental condition and/or the study hypothesis during data collection.*

Timing	*Indicate the start and stop dates of data collection. If there is a gap between collection periods, state the dates for each sample cohort.*
Data exclusions	*If no data were excluded from the analyses, state so OR if data were excluded, provide the exact number of exclusions and the rationale behind them, indicating whether exclusion criteria were pre-established.*
Non-participation	*State how many participants dropped out/declined participation and the reason(s) given OR provide response rate OR state that no participants dropped out/declined participation.*
Randomization	*If participants were not allocated into experimental groups, state so OR describe how participants were allocated to groups, and if allocation was not random, describe how covariates were controlled.*

Ecological, evolutionary & environmental sciences study design

All studies must disclose on these points even when the disclosure is negative.

Study description	This study evaluated SARS-CoV-2 contents and variants, determining lineages in wastewater from aircraft lavatories and Arlanda's airport sewage facilities and compared these with local population data to assess what information could be gained at each of these levels.
Research sample	From January to May 2023, weekly wastewater samples were collected from five locations in Stockholm: aircraft, Arlanda Airport Terminal 5, Måby station (covering airport region), Käppala WWTP, and Stockholm city. Stockholm city includes three main WWTPs: Bromma (18% of the population), Henriksdal (41%), and Käppala (33%). Bromma (Hässelby, Riksby and Järva) and Henriksdal (Sickla and Henriksdal) WWTPs have three and two inlets respectively. All airport wastewater goes to Käppala. Stockholm's samples were collected from week 1 to 22 of 2023 while aircraft samples from week 2 to 18, except for the 3rd and 11th weeks when flights were cancelled. A direct flight from China, with a maximum capacity of 312 passengers, was weekly monitored.
Sampling strategy	Flow-compensated samples were collected over 24 h from Monday to Tuesday every week, using stationary flow-proportional samplers SP5 B (MAXX Mess- und Probenahmetechnik GmbH, Germany), TP5 W (MAXX Mess- und Probenahmetechnik GmbH, Germany) or Efconomy (Efcon Water B.V., The Netherlands). The sample size was the number of samples obtained during the tested period, which varied from 17 to 22 weeks (one sample per week per location) . For each individual sample, two biological replicates were extracted for RNA analysis. Additionally, two qPCR quantifications (techinical replicates) were performed for each biological replicate. The standard deviation was calculated between the two biological replicates.
Data collection	SARS-CoV-2 contents were determined via reverse transcriptase quantitative polymerase chain reaction (RT-qPCR) using N3 primers targeting the nucleocapsid gene (N-gene). Pepper mild mottle virus (PMMoV) was also quantified in the samples and used for data normalization. Nuclease-free water and RNA extracted from tap water were included as negative controls for all qPCR reactions. SARS-CoV-2 DNA (2019-nCoV_N_Positive Control, IDT, Cat. 10006625), and a constructed plasmid containing the appropriate target for PMMoV (IDT, custom MiniGene 25-500 bp) were used as positive controls and to create the standard curves. A control sample of RNA from wastewater was used for quality control. Mariel Perez-Zabaleta, Isaac Owusu-Agyeman, and Ayda Kiyar were responsible for collecting SARS-CoV-2 data. The data was processed in Excel and shared among the group via email and OneDrive.<br>The sequencing data was obtained from Uppsala Genome Center (Science for Life Laboratory, Dept. of Immunology, Genetics and Pathology, Uppsala University). The samples were then reverse-transcribed with SuperScriptVILO cDNA synthesis (Thermo Fisher Scientific, Waltham, MA, USA) and the libraries were prepared using the AmpliSeq SARS-CoV-2 panel (Thermo Fisher Scientific) on two S5 540 chips. The libraries were barcoded and pooled into 32-plex and sequenced on the Ion S5XL system (Thermo Fisher Scientific). Processing of raw sequencing reads, and single nucleotide variant (SNV) calling was done through the Torrent Suit AmpliSeq SARS-CoV-2 pipeline according to the manufacturer's instructions. The resulting BAM files were used for post-processing with the Freyja pipeline, v.1.4.8. Carlo Berg and Luisa W. Hugerth were responsible for processing the data. "Freyja variants" were used to track SNVs against the Wuhan-2019-nCoV reference genome. Then, to convert the depth of each SNV into a likely relative abundance of Pango lineages, "Freyja demix" was used with barcode version 12_12_2023-00-48, a depth cutoff of 10x, a minimum relative abundance (eps) of 0.001 and only confirmed variants 22–24. Sequences are deposited at ENA under https://www.ebi.ac.uk/ena/browser/view/PRJEB61810.
Timing and spatial scale	Stockholm's samples were collected from week 1 to 22 of 2023 while aircraft and airport samples from week 2 to 18, except for the 3rd and 11th weeks when flights were canceled. Weekly monitoring were performed as recommended by the CDC (https://www.cdc.gov/nwss/sampling.html)
Data exclusions	No data was excluded from the analysis
Reproducibility	To ensure reproducibility, we included positive and negative controls in our qPCR reactions.<br>Negative Controls helped to verify that any detected signals are specific to the target of interest and not due to contamination or artifacts: Nuclease-free water and RNA extracted from tap water served as negative controls.<br>We used the following positive controls:<br>SARS-CoV-2 DNA (2019-nCoV_N_Positive Control, IDT, Cat. 10006625): This control confirms the functionality of our SARS-CoV-2 detection assay.<br>A constructed plasmid containing the appropriate target for PMMoV (IDT, custom MiniGene 25-500 bp): This control validates the PMMoV detection.<br>A control sample of RNA from wastewater: This ensures the quality and reliability of our wastewater samples.

Randomization	During the concentration and RNA extraction steps, the samples were randomized, resulting in two distinct groups. Group one consisted of the initial set of samples, including Henriskdal 1, Sickla 2, Hässelby 3, and so on. Group two, on the other hand, contained the duplicate samples.
Blinding	In the qPCR plates, the samples were once again randomized. During data acquisition, blinding procedures were rigorously followed to ensure that both data collectors and analysts remained unaware of the group assignments. This meticulous blinding process effectively minimized bias and facilitated an unbiased assessment of outcomes.

Did the study involve field work? [x] Yes [ ] No

Field work, collection and transport

Field conditions	Field trips were conducted to collect samples. Sampling took place during the winter and spring seasons, with temperatures ranging from approximately -10°C to 15°C. We used protective gear, including lab coats, gloves, and facemasks, to guard against potential infections.
Location	name of the sampling site, geographic location (latitude) and (longitude)<br>Malmö 55.63 13.04<br>Henriksdal inlet 59.31 18.11<br>Sickla inlet 59.31 18.11<br>Hässelby inlet 59.35 17.97<br>Järva inlet 59.35 17.97<br>Riksby inlet 59.35 17.97<br>Käppala 59.35 18.22<br>Aircraft N/A N/A<br>Terminal 5 59.65 17.93<br>Måby 59.63 17.89
Access & import/export	Sampling at the airport and on aircraft was only permitted with authorization from the Swedish authorities. As this study was mandated by the Public Health Authority of Sweden, they facilitated access and granted permission.
Disturbance	-

Reporting for specific materials, systems and methods

We require information from authors about some types of materials, experimental systems and methods used in many studies. Here, indicate whether each material, system or method listed is relevant to your study. If you are not sure if a list item applies to your research, read the appropriate section before selecting a response.

Materials & experimental systems

n/a	Involved in the study	
[x]	[ ]	Antibodies
[x]	[ ]	Eukaryotic cell lines
[x]	[ ]	Palaeontology and archaeology
[x]	[ ]	Animals and other organisms
[ ]	[x]	Clinical data
[x]	[ ]	Dual use research of concern
[x]	[ ]	Plants

Methods

n/a	Involved in the study	
[x]	[ ]	ChIP-seq
[x]	[ ]	Flow cytometry
[x]	[ ]	MRI-based neuroimaging

Antibodies

Antibodies used	No
Validation	-

Eukaryotic cell lines

Policy information about cell lines and Sex and Gender in Research

Cell line source(s)	-
Authentication	-

Mycoplasma contamination	-
Commonly misidentified lines (See ICLAC register)	-

Palaeontology and Archaeology

Specimen provenance	Not applicable for this research
Specimen deposition	Not applicable for this research
Dating methods	Not applicable for this research

☐ Tick this box to confirm that the raw and calibrated dates are available in the paper or in Supplementary Information.

Ethics oversight	Not applicable for this research

Note that full information on the approval of the study protocol must also be provided in the manuscript.

Animals and other research organisms

Policy information about studies involving animals; ARRIVE guidelines recommended for reporting animal research, and Sex and Gender in Research

Laboratory animals	Not applicable for this research
Wild animals	Not applicable for this research
Reporting on sex	Not applicable for this research
Field-collected samples	Not applicable for this research
Ethics oversight	Not applicable for this research

Note that full information on the approval of the study protocol must also be provided in the manuscript.

Clinical data

Policy information about clinical studies

All manuscripts should comply with the ICMJEguidelines for publication of clinical research and a completedCONSORT checklist must be included with all submissions.

Clinical trial registration	N/A
Study protocol	N/A
Data collection	Data was collected by the Swedish Health Authority (Folkhälsomyndigheten) from the authorized hospitals. The only data reported is SARS-CoV2 lineage from patient isolates, and no patient co-variables.
Outcomes	SARS-CoV2 lineage from patient isolates; no information on patient recovery or survival was taken

Dual use research of concern

Policy information about dual use research of concern

Hazards

Could the accidental, deliberate or reckless misuse of agents or technologies generated in the work, or the application of information presented in the manuscript, pose a threat to:

No	Yes	
☒	☐	Public health
☒	☐	National security
☒	☐	Crops and/or livestock
☒	☐	Ecosystems
☒	☐	Any other significant area

Experiments of concern

Does the work involve any of these experiments of concern:

No	Yes	
☒	☐	Demonstrate how to render a vaccine ineffective
☒	☐	Confer resistance to therapeutically useful antibiotics or antiviral agents
☒	☐	Enhance the virulence of a pathogen or render a nonpathogen virulent
☒	☐	Increase transmissibility of a pathogen
☒	☐	Alter the host range of a pathogen
☒	☐	Enable evasion of diagnostic/detection modalities
☒	☐	Enable the weaponization of a biological agent or toxin
☒	☐	Any other potentially harmful combination of experiments and agents

Plants

Seed stocks	Not applicable for this research
Novel plant genotypes	Not applicable for this research
Authentication	Not applicable for this research

ChIP-seq

Data deposition

☐ Confirm that both raw and final processed data have been deposited in a public database such as GEO.

☐ Confirm that you have deposited or provided access to graph files (e.g. BED files) for the called peaks.

Data access links<br>*May remain private before publication.*	*For "Initial submission" or "Revised version" documents, provide reviewer access links. For your "Final submission" document, provide a link to the deposited data.*
Files in database submission	*Provide a list of all files available in the database submission.*
Genome browser session<br>(e.g. UCSC)	*Provide a link to an anonymized genome browser session for "Initial submission" and "Revised version" documents only, to enable peer review. Write "no longer applicable" for "Final submission" documents.*

Methodology

Replicates	*Describe the experimental replicates, specifying number, type and replicate agreement.*
Sequencing depth	*Describe the sequencing depth for each experiment, providing the total number of reads, uniquely mapped reads, length of reads and whether they were paired- or single-end.*
Antibodies	*Describe the antibodies used for the ChIP-seq experiments; as applicable, provide supplier name, catalog number, clone name, and lot number.*
Peak calling parameters	*Specify the command line program and parameters used for read mapping and peak calling, including the ChIP, control and index files used.*

Data quality	*Describe the methods used to ensure data quality in full detail, including how many peaks are at FDR 5% and above 5-fold enrichment.*
Software	*Describe the software used to collect and analyze the ChIP-seq data. For custom code that has been deposited into a community repository, provide accession details.*

Flow Cytometry

Plots

Confirm that:

- [ ] The axis labels state the marker and fluorochrome used (e.g. CD4-FITC).
- [ ] The axis scales are clearly visible. Include numbers along axes only for bottom left plot of group (a 'group' is an analysis of identical markers).
- [ ] All plots are contour plots with outliers or pseudocolor plots.
- [ ] A numerical value for number of cells or percentage (with statistics) is provided.

Methodology

Sample preparation	*Describe the sample preparation, detailing the biological source of the cells and any tissue processing steps used.*
Instrument	*Identify the instrument used for data collection, specifying make and model number.*
Software	*Describe the software used to collect and analyze the flow cytometry data. For custom code that has been deposited into a community repository, provide accession details.*
Cell population abundance	*Describe the abundance of the relevant cell populations within post-sort fractions, providing details on the purity of the samples and how it was determined.*
Gating strategy	*Describe the gating strategy used for all relevant experiments, specifying the preliminary FSC/SSC gates of the starting cell population, indicating where boundaries between "positive" and "negative" staining cell populations are defined.*

- [ ] Tick this box to confirm that a figure exemplifying the gating strategy is provided in the Supplementary Information.

Magnetic resonance imaging

Experimental design

Design type	Not applicable for this research
Design specifications	*Specify the number of blocks, trials or experimental units per session and/or subject, and specify the length of each trial or block (if trials are blocked) and interval between trials.*
Behavioral performance measures	*State number and/or type of variables recorded (e.g. correct button press, response time) and what statistics were used to establish that the subjects were performing the task as expected (e.g. mean, range, and/or standard deviation across subjects).*

Acquisition

Imaging type(s)	*Specify: functional, structural, diffusion, perfusion.*
Field strength	*Specify in Tesla*
Sequence & imaging parameters	*Specify the pulse sequence type (gradient echo, spin echo, etc.), imaging type (EPI, spiral, etc.), field of view, matrix size, slice thickness, orientation and TE/TR/flip angle.*
Area of acquisition	*State whether a whole brain scan was used OR define the area of acquisition, describing how the region was determined.*

Diffusion MRI ☐ Used ☐ Not used

Preprocessing

Preprocessing software	*Provide detail on software version and revision number and on specific parameters (model/functions, brain extraction, segmentation, smoothing kernel size, etc.).*
Normalization	*If data were normalized/standardized, describe the approach(es): specify linear or non-linear and define image types used for transformation OR indicate that data were not normalized and explain rationale for lack of normalization.*

Normalization template | *Describe the template used for normalization/transformation, specifying subject space or group standardized space (e.g. original Talairach, MNI305, ICBM152) OR indicate that the data were not normalized.*

Noise and artifact removal | *Describe your procedure(s) for artifact and structured noise removal, specifying motion parameters, tissue signals and physiological signals (heart rate, respiration).*

Volume censoring | *Define your software and/or method and criteria for volume censoring, and state the extent of such censoring.*

Statistical modeling & inference

Model type and settings | *Specify type (mass univariate, multivariate, RSA, predictive, etc.) and describe essential details of the model at the first and second levels (e.g. fixed, random or mixed effects; drift or auto-correlation).*

Effect(s) tested | *Define precise effect in terms of the task or stimulus conditions instead of psychological concepts and indicate whether ANOVA or factorial designs were used.*

Specify type of analysis: ☐ Whole brain ☐ ROI-based ☐ Both

Statistic type for inference
(See Eklund et al. 2016) | *Specify voxel-wise or cluster-wise and report all relevant parameters for cluster-wise methods.*

Correction | *Describe the type of correction and how it is obtained for multiple comparisons (e.g. FWE, FDR, permutation or Monte Carlo).*

Models & analysis

n/a	Involved in the study
☐	☐ Functional and/or effective connectivity
☐	☐ Graph analysis
☐	☐ Multivariate modeling or predictive analysis

Functional and/or effective connectivity | *Report the measures of dependence used and the model details (e.g. Pearson correlation, partial correlation, mutual information).*

Graph analysis | *Report the dependent variable and connectivity measure, specifying weighted graph or binarized graph, subject- or group-level, and the global and/or node summaries used (e.g. clustering coefficient, efficiency, etc.).*

Multivariate modeling and predictive analysis | *Specify independent variables, features extraction and dimension reduction, model, training and evaluation metrics.*
